# An Analysis of the Content of Metalloproteinases in the Intestinal Wall of Patients with Crohn’s Disease

**DOI:** 10.3390/life13102013

**Published:** 2023-10-05

**Authors:** Grzegorz Chrzanowski, Grzegorz Pasternak, David Aebisher, Klaudia Dynarowicz, Angelika Myśliwiec, Dorota Bartusik-Aebisher, Barbara Sosna, Grzegorz Cieślar, Aleksandra Kawczyk-Krupka, Rafał Filip

**Affiliations:** 1 Department of Biology, College of Natural Sciences, University of Rzeszów, 35-310 Rzeszów, Poland; gchrzanowski@ur.edu.pl; 2 Department of General Surgery, Provincial Clinical Hospital No. 2 in Rzeszów, 35-301 Rzeszów, Poland; g.past.gp@gmail.com; 3 Department of Photomedicine and Physical Chemistry, Medical College of University of Rzeszów, University of Rzeszów, 35-310 Rzeszów, Poland; 4 Center for Innovative Research in Medical and Natural Sciences, Medical College of the University of Rzeszów, 35-310 Rzeszów, Poland; kdynarowicz@ur.edu.pl (K.D.); amysliwiec@ur.edu.pl (A.M.); 5 Department of Biochemistry and General Chemistry, Medical College of University of Rzeszów, University of Rzeszów, 35-310 Rzeszów, Poland; dbartusikaebisher@ur.edu.pl; 6 Department of Internal Medicine, Angiology and Physical Medicine, Center for Laser Diagnostics and Therapy, Medical University of Silesia in Katowice, Batorego 15 Street, 41-902 Bytom, Poland; barbara.sosna@hotmail.com (B.S.); gcieslar@sum.edu.pl (G.C.); akawczyk@gmail.com (A.K.-K.); 7 Department of Internal Medicine, Medical College of University of Rzeszów, University of Rzeszów, 35-310 Rzeszów, Poland; rfilip@ur.edu.pl

**Keywords:** Crohn’s disease, metalloproteinases, in vitro studies

## Abstract

One of the inflammatory bowel diseases is Crohn’s disease. Although this term has been used in the medical community since 1932, a significant increase in the number of publications occurs at the end of the 20th century and the beginning of the 21st century. Crohn’s disease is a disease that cannot be fully cured. In many cases, it is chronic, i.e., recurrent. All preventive and therapeutic measures taken by doctors are aimed at inhibiting the development of the disease and minimizing the occurrence of any potential “side effects” resulting from the developing disease. One of the diagnostic methods is the qualitative and quantitative determination of metalloproteinases in inflammatory tissues and in the blood. The aim of the study was the quantitative and qualitative determination of metalloproteinases in inflammatory bowel tissues in patients diagnosed with Crohn’s disease. The in vitro study was performed on surgical tissues from patients diagnosed with Crohn’s disease. The results show that in inflammatory tissues the concentration of metalloproteinases -3, -7, -8, -9 was higher compared to tissues taken from the resection margin without signs of inflammation, defined as healthy. The experiment confirmed that the biochemical test, which is the determination of metalloproteinases in tissues, is a useful diagnostic tool to differentiate inflammatory from non-inflammatory tissues.

## 1. Introduction

Any developing inflammatory diseases (including Crohn’s disease) cause short-term or permanent changes in the cellular level. Each inflammatory change has a destructive effect on metabolic processes and the activity of receptors or enzymes [1]. Crohn’s disease belongs to the group of inflammatory bowel diseases, i.e., a set of chronic inflammatory diseases [2,3,4,5,6,7,8,9,10,11,12]. This disease cannot be fully cured, however, using appropriate diagnostic and therapeutic procedures, emerging inflammatory changes can be controlled [13]. This disease can occur at any age. However, it is most often diagnosed between the ages of 15 and 30, i.e., in young people in the second and third decades of life [14]. People over 65 may also develop some symptoms. Based on the Agency for Health Technology Assessment and Tariffs of 12 June 2019, it is estimated that in the European Union, 40 to 50 people per 100,000 inhabitants suffer from Crohn’s disease [15]. It is worth noting that more and more often this disease affects children before the age of 5 [16]. This disease does not depend on gender, men and women are affected to the same extent. The incidence of Crohn’s disease is constantly increasing [17]. In the years 1955–1995, in selected European Union countries, the incidence rates doubled [18]. The disease is most common in developed countries. According to experts, a significant increase in morbidity is conditioned by environmental factors associated with a significant improvement in the economic status of the countries of Western Europe, Central and Eastern Europe and North America. These factors may include food modification and factors linked to economic progress. In Crohn’s disease, the physiology and functioning of the epithelial barrier are inhibited due to the correlation of various factors, such as: environment, genetic susceptibility or intestinal microbiota [19]. 

Metalloproteinases (MMPs) play an important role in inflammation and cancer progression by promoting cancer cell growth, migration, invasion, formation metastases and new blood vessels. The basic function of metalloproteinases is to participate in physiological and pathological processes of reconstruction of extracellular matrix components and their degradation. The secretion and activity of metalloproteinases are increased in almost all types of human cancers and correlate with the stage of advancement, greater invasiveness, the ability to metastasize, and with shorter survival.

To promote rapid and straightforward detection of MMP activity, many methods have been developed based on highly sensitive fluorescence measurement using fluorescent dyes [20], or nanomaterials [21]. To date, a zymographic technique has long been used as the standard method for assaying MMP activity, which easily detects the activities of different MMPs by degrading the preferential substrate based on the molecular weight [22].

MMPs play an important role in inflammation and cancer progression by promoting cancer cell growth, migration, invasion, formation metastases and new blood vessels. The basic function of metalloproteinases is to participate in physiological and pathological processes of reconstruction of extracellular matrix components and their degradation. The secretion and activity of metalloproteinases are increased in almost all types of human cancers and correlate with the stage of advancement, greater invasiveness, the ability to metastasize, and with shorter survival.

The aim of this work was to investigate tissue metalloproteinases as markers of recurrence of Crohn’s disease in intestinal biopsies from surgical patients from postoperative preparations and obtained by colonoscopy.

## 2. Material and Methods 

### 2.1. Large Intestine Tissue Samples 

**Figure 1:**
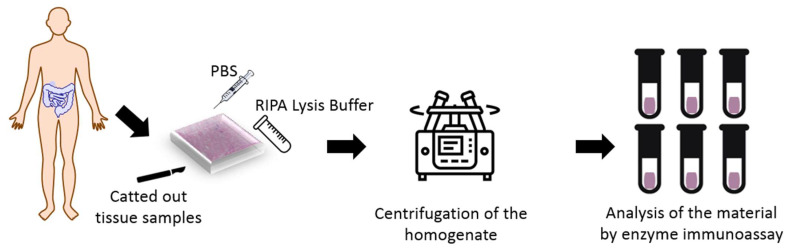
Test procedure.

The study was conducted in the Department of General Surgery, Provincial Clinical Hospital No. 2 in Rzeszów in the years 2018–2020. The experimental groups included 31 patients aged 23 to 70 years, with an average age of 40.4 years. Large intestine tissue samples were taken by resection selected section of the intestine or during colectomy. A total of 41 samples were used. The collected of samples had a volume of 13 × 7 × 8 mm. Figure 1 shows procedure of experimental test. 

Characteristic of the study population are presented in Table 1. 

In turn, Table 2 presents detailed characteristics of the research group (form and type of treatment as well as the date of reoperation. A test for the presence of *Escherichia coli* bacteria was also performed. Some patients tested positive. Patients infected with *Escherichia coli* have experienced symptoms such as diarrhea and gastrointestinal infection.

Patients diagnosed with Crohn’s disease received pharmacological treatment, such as: azathioprine, 5-ASA, Infliximab, Mercaptopurine, adalimumab, steroids, Ciprofloxacin, methipred.

The entire sampling process was carried out in the Department of General Surgery, Provincial Clinical Hospital No. 2 in Rzeszów. The work presented here is approved by RESOLUTION No. 2018/06/04 of the Bioethics Committee of the University of Rzeszów. 

### 2.2. Procedure of Preparation Samples

Sick and healthy tissues were catted out from the large intestine of the patients with Crohn’s disease and healthy patients. There were in experimental groups, thirty-one sick patients and ten tissue with the healthy digestive tract. Those parts of tissues were treated with liquid nitrogen and immediately were frozen and stored at −80 °C until analyses were performed. 

25 to 45 mg of the tissue piece was rinsed in ice-cold PBS buffer to remove the excess blood, dried with filter paper, and weighed again. Fragmented tissues were homogenized with Ripa Lysis buffer on the ice at 10:1 (10 μL chilled RIPA Buffer per milligram of tissue). The lysis buffer contained 1% sodium deoxycholate, 0.1% SDS, and protease and phosphatase inhibitors added immediately before use. Then the homogenate was centrifuged for 10 min at 10,000× *g*. The supernatant was collected, and four metalloproteinases—MMP 3, MMP 7, MMP 8 and MMP 9 were analyzed by Enzyme-linked Immunosorbent Assay using SEA101HU, SEA102Hu, SEA103Hu and SEA553Hu kits (Cloud-Clone Corp., Kata, TX, USA), respectively. All chemical analyzes were performed in triplicate. 

### 2.3. Characteristics of Enzyme-Linked Immunosorbent Assay

Application of the Enzyme-linked Immunosorbent Assay using SEA101HU, SEA102Hu, SEA103Hu and SEA553Hu kits (Cloud-Clone Corp., Kata, TX, USA) enabled the determination of 4 metalloproteinases in tissue samples in vitro. Accordingly to manufactures SEA101HU kit is the assay with high sensitivity and excellent specificity for detection of MMP3. The detection range of SEA101HU kit is in range of 31.2–2000 pg/mL and the minimum detectable dose for this kit is typically less than 13.1 pg/mL. The detection range for the SEA102HU kit, used to detect MMP7 is in range of 0.156–10 ng/mL and the minimum detectable dose for this kit is typically less than 0.063 ng/mL. For the detection of MMP8 was used SEA103HU kit with the detection range of 78–5000 pg/mL and the minimum detectable dose of this kit is typically less than 28 pg/mL. The MMP9 was detected with the kit SEA553HU characterized by detection range of 0.312–20 ng/mL and the minimum detectable dose less than 0.125 ng/mL. All reagents, samples and standards listed in Table 3 were necessary to be prepared to perform assay. Each assay kit included reagents listed in Table 3. 

The preparation of kit to perform assay in each well was done in the following steps: (1) 100 µL of standard or sample was added and incubated for 1 h at 37 °C; (2) 100 µL of prepared detection Reagent A was added and incubate for 1 h at 37 °C; (3) aspirated and washed 3 times; (4) 100 µL prepared Detection Reagent B was added and incubated for 30 min at 37 °C; (5) aspirated and washed 5 times; (6) 90 µL of substrate solution was added and incubate 10–20 min at 37 °C and 50 µL of stop solution was added and reading was performed at 450 nm immediately. All chemical analyzes were performed in triplicate. Metalloproteinase content was expressed as mean ± standard deviation.

Absorbance at 450 nm was measured using an ELISA plate reader (Tecan Infinite 200 PRO). The presence or absence of MMP was determined by comparing the absorbance of samples to negative (healthy sample) and positive (sick sample) controls. A standard curve was generated by preparing 7 mixtures of the negative and positive controls (0, 5, 10, 25, 50, 75 and 100%).

Zymographic technique is process with protein unfolding and refolding steps and determining the level of MMP activity is difficult, as MMP exists in its complex form with tissue inhibitor of metalloproteinase (TIMP) in the real sample; moreover, sodium dodecyl sulfate (SDS) dissociates TIMPs from MMPs during electrophoresis. 

Gelatinolytic zymography was performed as previously described and the amounts of MMPs were quantified using scanning densitometry with an image analysis software [23]. Electrophoresis was performed and, after a brief wash with water, SDS was removed from the gel by incubation with 2% Triton X-100/PBS solution. Gelatinolytic activities were developed in a buffer containing 5 mM CaCl2, 150 mM NaCl, and 50 mM Tris at 37 °C for 16 h and then visualised by staining the gel with Coomassie Blue R-250 [23].

### 2.4. Statistical Analysis 

Differences in the metalloproteinases levels in tissues of sick and healthy patients were calculated by the *t*-Student test at *p* < 0.05, using Statistica 13.3 software (TIBCO Software Inc., Palo Alto, CA, USA).

## 3. Results

**Figure 2:**
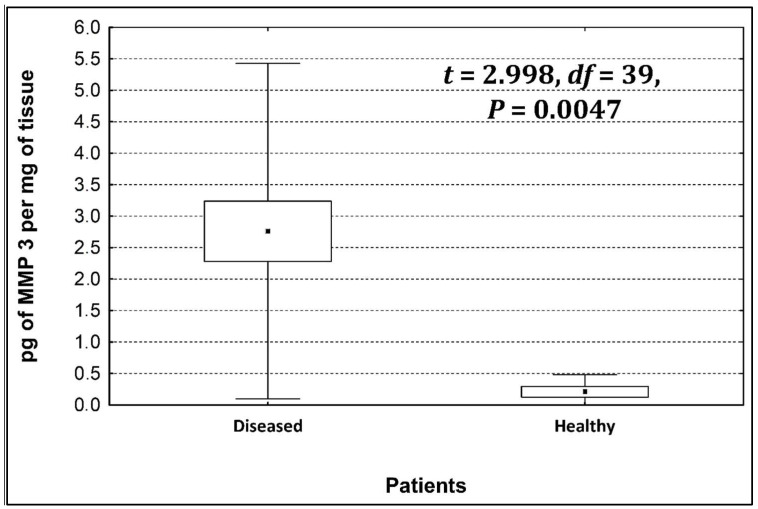
The content of metalloproteinase 3 within the large intestine of patients.

**Figure 3:**
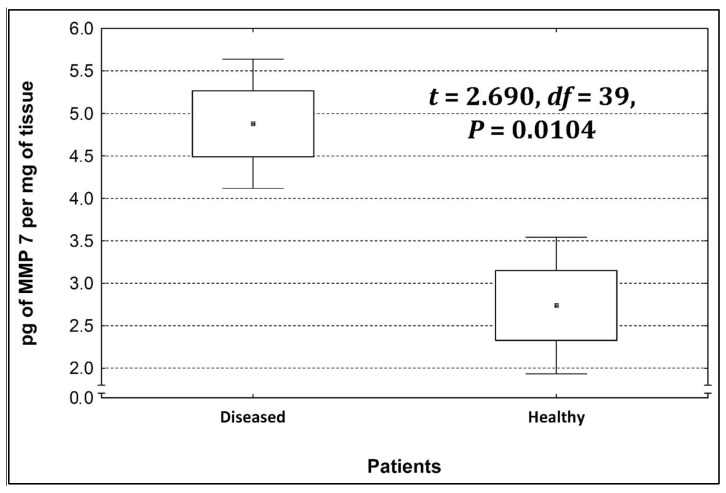
The content of metalloproteinase 7 within the large intestine of patients.

**Figure 4:**
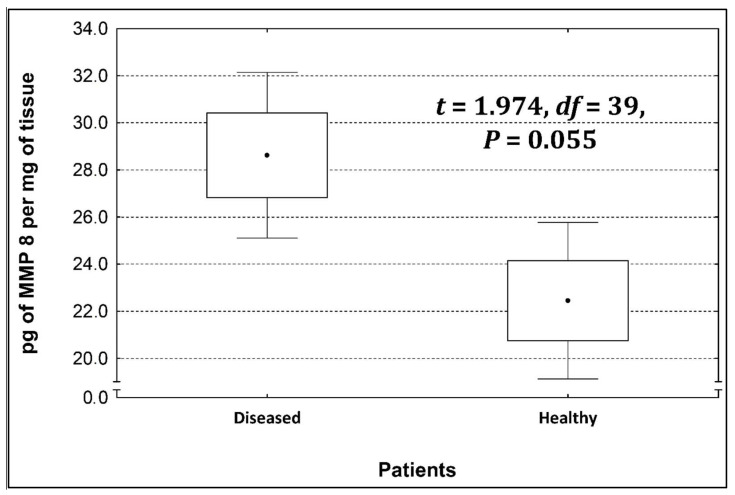
The content of metalloproteinase 8 within the large intestine of patients.

**Figure 5:**
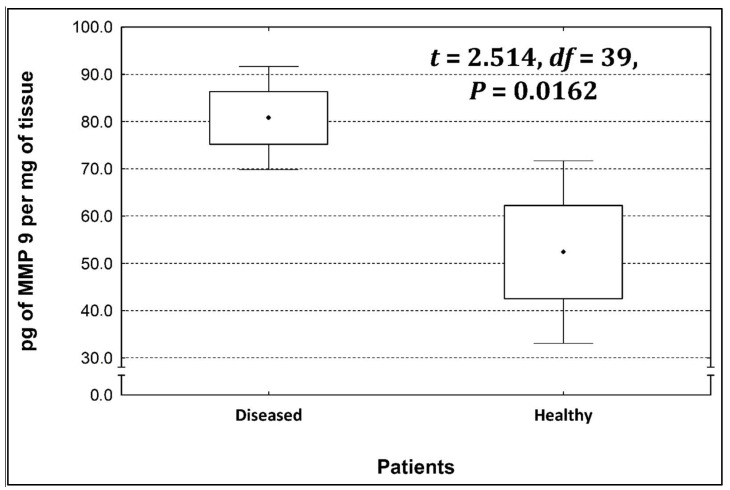
The content of metalloproteinase 9 within the large intestine of patients.

Four metalloproteinases were found, and their content was determined within the large intestine of patients; there were metalloproteinases 3, 7, 8, and 9 (Figure 2, Figure 3, Figure 4 and Figure 5). The pathologically altered tissue contained higher levels of all tested metalloproteinases than healthy ones. However, statistical differences between their content were proven for MMPs 3, 7, and 9. It was found that metalloproteinase nine (MMP-9, Figure 5) showed the highest amount (80.8 pg per mg of tissue) amongst those tested proteinases. The lowest metalloproteinases content within sick patient tissues was shown for proteinase three (2.8 pg) and seven (4.9 pg).

Table 4 shows the concentrations of metalloproteinases in diseased and healthy tissue.

The ratio of MMP’s concentration in sick tissue to MMP’s concentration in healthy tissues were 13.3:1; 1.78:1; 1.26:1; 1.53;1 for MMP-3, MMP-7, MMP-8, and MMP-9, respectively. In the paper by Kirkegaard et al. MMP-9 positive cells was significantly (2–20-fold) higher than those for MMP-1, MMP-7, or both [24]. In our study we received ratio of MMP-9: MMp7 = 16.48:1. On the one hand, they are a prognostic factor, and on the other, they are a factor monitoring the effectiveness of disease therapy.

Increased expression of MMP-9 in colorectal cancer tissue has been demonstrated, correlating with the stage of disease advancement, greater invasiveness and shorter survival time of patients (2, 19, 25). Their expression was monitored, among others, in osteosarcomas, mast cell tumors and lymphomas in both humans and animals. Their activity was correlated with the degree of malignancy of tumors and the tendency to metastasize (2, 17, 19) [25,26].

The conducted research has shown that the activity of MMP-2 and -9 is higher in tissues affected by cancer compared to healthy tissues. The results obtained in studies on mast cell tumors show that in this case MMP-9 has greater diagnostic and prognostic significance, while the activity of MMP-2 varied depending on the degree of tumor differentiation, but not to such a significant extent. Greater expression is also visible in tumors of higher malignancy [27].

**Figure 6:**
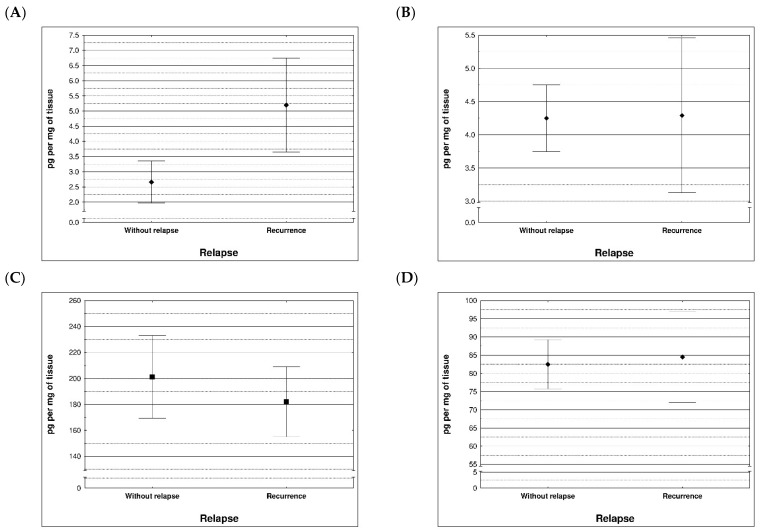
Content of metalloproteinases within tissues of patients with and without recurrence of Crohn disease; (**A**) MMP 3 (*p* = 0.0296), (**B**) MMP 7 (*p* = 0.710), (**C**) MMP 8 (*p* = 0.396), (**D**) MMP 9 (*p* = 0.710).

**Figure 7:**
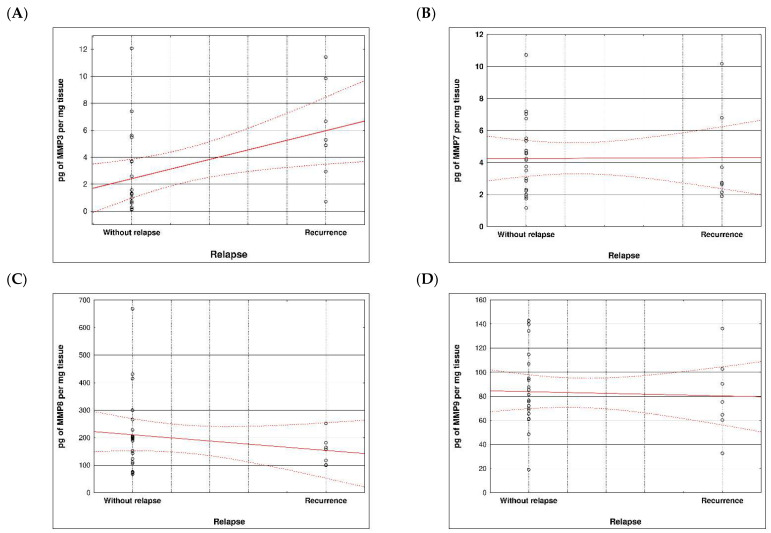
Correlation between recurrence of Crohn disease and metalloproteinases content within sick tissues of patients; (**A**) MMP 3, (**B**) MMP 7, (**C**) MMP 8, (**D**) MMP 9.

During examination in the large intestine, the presence of four metalloproteinases was found in the examined patients, the content of which was determined in the large intestine. (MMP 3, 7, 8 and 9). The statistical model constructed on the basis of chemical analyses demonstrated two variables (metalloproteinase 3 and metalloproteinase 8) significantly influenced the possibility of Crohn recurrence (Figure 6 and Figure 7 and Table 5 and Table 6).

Concerning the MMPs serum expression performed by zymography, analysis of the results showed that the concentration of metalloproteinases -3, -7, -8, -9 was higher compared to tissues taken from the resection margin without signs of inflammation, defined as health higher concentrations of in patients diagnosed with to the group of healthy samples.

Specifically, the mean serum MMP-9 concentration was 20 fold higher in patients than in control group. Also in this case the difference was statistically significant (*p* = 0.05).

## 4. Discussion 

The key role in cell-based diagnostics is played by the analysis of metalloproteinases from the group of stromelysins. MMP-3 and MMP-10 are the main enzymes indicating the development of Crohn’s disease [28]. Their activity is increased, e.g., in developing fistulas of Crohn’s disease [29]. In turn, the activity of MMP-9 metalloproteinases is higher in patients with Crohn’s disease, but it is not the main factor of the disease. Its activity can be inhibited by ramipril, used in the form of an inhibitor, i.e., a substance that inhibits the activity of a given enzyme. Elevated content of MMP-1 in the blood serum in the case of inflammatory bowel disease indicates an increase in the number of cytokines, i.e., proteins that play a key role in the immune response. Indirectly, they may indicate an ongoing inflammation in the patient’s body. Metalloproteinases can stimulate or inhibit the processes of action of various receptors and cells. In the literature, they are defined as enzymes that destroy the structural components of the extracellular matrix [30,31]. Also in the case of pediatric patients diagnosed with Crohn’s disease, the concentration of MMP-3 and MMP-9 increases with the activity of the disease [32]. Metalloproteinases correlate with inflammation, giving a signal about the phase of the disease [32]. The MMP-9 assay is also useful in the dormant phases of Crohn’s disease. In the case of an asymptomatic period, the doctor can assess the inflammatory bowel condition using a biochemical test [33]. A similar observation was made by Meijer et al., who also assessed inflamed tissues by biochemical analysis of mucosal metalloproteinase activity in patients with Crohn’s disease. They characterized markers MMP-1, -2, -3 and -9. Increased activity of all 4 metalloproteinases was demonstrated in inflammatory tissues, which may have contributed to changes in tissue morphology and physiology [34]. In addition, MMP-7 is a biomarker of Crohn’s disease as a differentiating marker for inflammatory tissues. In a study by Rath et al. increased mRNA levels of MMP-2, MMP-7, and MMP-13 have been reported in Crohn’s disease biopsy specimens. MMP-2 and MMP-9 indicated increased protein secretion [35]. Jakubowska, et al.; based on their research, they also observed an increase in the concentration of MMP-2, MMP-7 and MMP-9. Researchers suggest that the characterized metalloproteinases may be a potential therapeutic target, and the use of their inhibitors may significantly reduce the progression of Crohn’s disease [36]. In studies conducted in pediatric patients, serum MMP-7 reflected disease activity [37,38]. 

MMPs are a large group of zinc-dependent proteolytic enzymes that are involved in the degradation and remodeling of the extracellular matrix by cleaving specific elements [39]. At the level of gene transcription, many cytokines and growth factors are factors that stimulate MMP expression, including: interleukin 1 (IL-1), interleukin 6 (IL-6), tumor necrosis factor a (TNF-a), epidermal growth factor (EGF), platelet-derived growth factor factor—PDFG), basic fibroblast growth factor (bFGF), hepatocyte growth factor (HGF) and others, and CD40 antigen.

According to the literature, increased MMP expression plays various roles in the pathogenesis, the cycles of acute inflammation and resolution, and chronic processes such as fibrosis and fistula forms in Crohn’s disease. The function of both beneficial and unfavorable MMPs has not yet been well studied. However, this knowledge has been consolidated for about 6 years [40]. However, in terms of the regenerative role of MMPs, they have not yet been sufficiently studied in IBD [41].

The incidence of inflammatory bowel diseases has been increasing worldwide for about 20 years. Some Western countries, such as Canada, predict an increase of nearly 33.4% between 2015 and 2025 [39]. As is known, the etiology of the disease is still unexplored at an adequate level, but many studies in this field have identified MMPs as risk factors for the development and progression of diseases due to proteolytic regulation or modulation of transcription factors [42,43,44,45,46,47,48,49,50,51,52,53].

Metalloproteinases are activated following interactions in the cell-cell and cell-ECM areas or in response to pro-inflammatory cytokines that are widely expressed [54,55,56].

MMPs are involved in the modulation of the pathogenesis of Crohn’s disease, and cytokines involved in inflammatory processes, developing in the intestine, have the ability to increase the level of MMPs. For example, TNF-α and bradykinin are able to induce MMP-3 expression through a signaling cascade [57].

Metalloproteinases are important in many human diseases, but no synthetic broad-spectrum MMP inhibitor has been adequately tested in clinical trials for both pro-cancer and anti-cancer effects of MMPs in cancer [58]. MMPs (MMP-2, MMP-9, MMP-14) are able to damage the capillary layer and at the same time promote the exosmosis of cancer cells. MMP-9 may downregulate the IL receptor found on the surface of T lymphocytes, and may suppress immunity and promote cancer development [58,59]. In the case of MMP-8, it can directly inhibit tumor metastasis. 

Metalloproteinases play a role in such processes as: (1) immune response, (2) angiogenesis, (3) influence on epithelial barrier function, (4) inflammation-induced fibrosis, (5) carcinogenesis [60].

In inflammatory bowel diseases, a self-perpetuating “vicious circle” of unfavorable events has been described, causing an increase in the inflammatory process in the wall of the affected intestine. Bacteria penetrating the epithelial barrier into the intestinal wall matrix cause the release of the chemokine CXCL-8, which has a chemotactic effect on neutrophils, causing them to migrate to the site of infection. Neutrophils produce MMP-8 and MMP-9. MMP-8 and MMP-9 metalloproteinases together with propylene endopeptidase are involved in collagen degradation. The product of this process is the tripeptide: proline-glycine-proline (PGP). PGP molecules have a chemotactic effect on neutrophils, intensifying the process of neutrophil influx—a self-perpetuating vicious circle is created [61].

MMP-7 metalloproteinase activates alpha-defensin, which in turn modulates and reduces the activity of IL1, thereby reducing the intensity of the inflammatory process [62].

MMP-7 increases the expression of KC and MIP-2 chemokines, which have a chemotactic effect on neutrophils [63]. According to Deleon-Pennell, et al., MMP-9 can also increase chemokine activity [64].

TNF-α is one of the main pro-inflammatory factors (its level increases in the blood, intestinal mucosa and stool in patients). MMP-13 has been shown to be an activator of TNF-α [65].

The process of angiogenesis is believed to play a fundamental role in the course of inflammatory bowel diseases. Increased secretion of MMP-1, MMP-3, MMP-9 by endothelial cells affects various stages of angiogenesis. Metalloproteinases can play an opposite role in the process of angiogenesis, on the one hand, they activate proangogenic factors, on the other hand, antiangiogenic factors—by affecting the synthesis of angiostatin. MMPs facilitate the formation of new vessels by remodeling the ECM, which enables the migration of endothelial cells [66]. The degradation of collagen XVIII and plasminogen by selected metalloproteinases may stimulate the production of endostatin and angiostatin (anti-angiogenesis inhibiting factors) [67].

MMP-9 has the ability to activate bound forms of the vascular endothelial growth factor (VEGF-a), which intensifies and accelerates angiogenesis [68,69].

In experiments on mice, an increase in the level of MMP-9 leads to an increase in the concentration of endostatin, which causes an anti-angiogenic effect [70]. It is explained that the resultant of pro- or anti-angiogenic activity of metalloproteinases depends on the microenvironment of the extracellular matrix on which these enzymes act.

The integrity of the intestinal epithelial barrier is essential in maintaining homeostasis. Penetration of bacteria through the epithelial barrier into the lamina propria matrix of the mucosa may cause a local inflammatory response through the release of inflammatory mediators including metalloproteinases. Overexpression of MMP-9 reduces the differentiation of goblet cells in the intestinal mucosa [71], and through the Notch receptor, reduces the expression of mucin 2 [72]. Reduced production of mucin 2 (MUC 2) weakens the protective mucin barrier on the surface of the intestinal epithelium, which may lead to increased adhesion of Salmonella typhimurium bacteria to enterocytes [73]. The level of MMP-7 affects the healing of ulcers in the intestinal mucosa [74,75,76].

Active metalloproteinases present in the extracellular space can be inhibited by natural inhibitors Active metalloproteinases present in the extracellular space can be inhibited by natural tissue inhibitors, proteins with a molecular weight between 21–34 kDa, with which MMPs form non-covalent complexes in a 1:1 ratio. Currently, four naturally occurring TIMPs are known: TIMP-1, TIMP-2, TIMP-3, TIMP-4. Two of them, TIMP-1 and TIMP-2, show high affinity for gelatinases [77,78]. Of all four tissue MMP inhibitors, only TIMP-2 is a constitutively expressed protein. Endogenous MMPs inhibitors bind non-selectively to the enzyme, blocking the access of the substrate to the active site.

TIMPs differ slightly in specificity. TIMP-2, unlike TIMP-1, has a greater affinity for membrane MMPs (MT-MMP). It also has the ability to bind to the active and inactive form of MMP-2, contributing to the activation of pro-MMP-2. TIMP-1 can combine with MMP-9 and pro-MMP-9, but the significance of the formation of the pro-MMP-9/TIMP-1 complex is not fully explained [77]. TIMP-1, TIMP-2 and TIMP-4 are secreted in soluble form into the blood, while TIMP-3 is bound to ECM proteins. The source of TIMPs are various types of cells. The main role is assigned to vascular smooth muscle cells, macrophages and platelets. Among other endogenous substances, α2-macroglobulin present in serum also has the ability to inhibit the activity of MMPs [79].

### Future Research Directions

Over recent years, knowledge about the molecular biology of MMPs in inflammatory bowel diseases has been expanded. Biomarkers are analyzed based on in vitro and in vivo tests [80]. Currently, it is recognized that the regulated expression of MMPs plays a key role in the pathogenesis and characteristics of inflammation. The multifunctionality of MMPs has not yet been sufficiently researched, therefore further research is needed to analyze the role of MMPs in the pathogenesis of various functions. Currently, research is also needed to evaluate and analyze the correlation between inflammation and MMP levels. Precise determination of this relationship may limit the use of invasive screening tests using colonoscopy. The use of MMP biomarker diagnostics will be helpful in determining disease recurrence. Additionally, it would reduce the need for colonoscopy [81].

## 5. Conclusions

Some metalloproteinases are characterized by increased activity not at the beginning of the ongoing inflammation, but in the advanced stage of the disease. MMP-1, MMP-3, MMP-7, MMP-8 and MMP-9 are biomarkers of Crohn’s disease. Their presence and increased value inform about the emerging, ongoing inflammation. Therefore, in the case of suspicion of Crohn’s disease, the supervising staff reaches for yet another diagnostic tool, which is the biochemical determination of inflammatory metalloproteinases. 

Our future research will be directed to design and incorporating a positive and a negative control experiments for comparison with the tested metalloproteinases e.g., using C-reactive protein (CRP). 

The significant differences can be noted between the metalloproteinase levels obtained from healthy and sick tissues. This possibility to detect concentrations of metalloproteinases can be useful to predict early detection of inflammatory diseases. Metalloproteinases, through complex mechanisms that involve the induction of multiple signaling pathways, are very important in the pathway from any precancerous lesion or polyp to an advanced stage. Most metalloproteinase levels are increased in colitis. Therefore, testing the level of selected metalloproteinases can be used to predict the condition and development of inflammatory diseases.

## Figures and Tables

**Table 1:** Characteristics of the study group.

Tissue Type	Gender Females Males		Age (Years) Range Mean		Smoker Non-Smoker		Type of Procedure Resection Colectomy		The Number of Complications
Crohn’s disease patient (sick tissue)	13	18	23–70	40.4	6	25	30	1	10
Control tissue (healthy tissue)	Healthy tissues are the margin of tissue collected during the procedure without any signs of disease								

**Table 2:** Detailed characteristics of the research group (form and type of treatment as well as the date of reoperation.

Patient Number	Type of Treatment	Reoperation (Year, Type)
1	Azathioprine, 5-ASA	2011, Subtotal colectomy
2	Azathioprine, 5-ASA	2015, Subtotal colectomy
3	Infliximab, Adalimumab, Mercaptopurine, Encorton	2014, ileal resection and right hemicolectomy
4	Azathioprine, 5-ASA	
5	Azathioprine, 5-ASA	
6	Solu-Medrol, Encorton	
7	Ciprofloxacin, Metronidazole, Steroid therapy	
8	Immunosuppression, Asamax, Encorton, Hydrocortison, Proxacin, Metronidazole	
9	Solu-Medrol, Asamax, Cipropol, Metronidazole	
10	Azathioprine, Solu-Medrol, Cipronex, Metronidazole	
11	Azathioprine, Asamax, Proxacin, Metronidazole	2003, sigmoid resection
12	Proxacin, Metronidazole, Corhydron	
13	Azathioprine, Encorton	
14	Azathioprine, Solu-Medrol, Tazocin, Metronidazole	
15	Azathioprine, Ciprofloxacin, Metronidazole	2018, ileocecal resection
16	Azathioprine, Pentasa, Biotraxon, Metronidazole	2015, left hemicolectomy 2018, rectal resection
17	Pentasa, Proxacin, Metronidazole	
18	Pentasa, Proxacin, Metronidazole	
19	Mercaptopurine, Encorton, Hydrocortisone	
20	Azathioprine, Asamax, Biotraxon, Metronidazole, Infliximab	
21	Azathioprine, Tazocin	
22	Azathioprine, Proxacin, Metronidazole	
23	Azathioprine, Asamax, Encorton, Proxacin, Metronidazole	
24	Azathioprine, Solu-Medrol, Metypred (methylprednisolone)	
25	Azathioprine, Mesalazine, Encorton, Biotraxon, Metronidazole	
26	Azathioprine, Amoksiklav, Biotraxon, Metronidazole	
27	Solu-Medrol, Proxacin, Metronidazole	
28	Infliximab, Adalimumab, Hydrocortison, Proxacin, Metronidazole	
29	Cipronex, Metronidazole, Modulen IBD	
30	Infliximab, Azathioprine	
31	Azathioprine, Amoksiklav, Biotraxon, Metronidazole	

**Table 3:** Assay kit.

Assay Kit
Pre-coated, ready to use 96-well strip plate
Standard and Standard Diluent
Detection Reagent A and Assay Diluent A
Detection Reagent B and Assay Diluent B
TMB Substrate and Stop Solution
Wash Buffer (30× concentrate)

**Table 4:** The concentrations of metalloproteinases.

MMPs	Sick Tissue (n = 30) [pg per mg Tissue]	Healthy Tissue (n = 10) [pg per mg Tissue]	*p*-Value
**MMP-3**	2.8 ± 2.6	0.21 ± 0.25	0.0047 *
**MMP-7**	4.9 ± 0.8	2.75 ± 0.85	0.0104 *
**MMP-8**	28.3 ± 2.8	22.4 ± 3.5	0.055
**MMP-9**	80.8 ± 10.8	52.5 ± 18.5	0.0162 *

*—statistically significant result *p* < 0.05.

**Table 5:** Best model testing the effects of variables on Crohn disease recurrence.

Variables	Parameter Estimate	SE	*F*	*p*
MMP 3	0.087	0.022	14.58	0.0002
MMP 7	0.032	0.032	1.03	0.309
MMP 8	−0.002	0.001	6.04	0.014
MMP 9	0.001	0.002	0.108	0.742

**Table 6:** Model selection using Akaike’s information criterion (AIC) to determine the effect of tested variables on Crohn disease recurrence.

Variables	AIC	Δ(AIC)	wt.(AIC)	k
MMP3 + MMP8	27.48	0.00	0.374	3
MMP3 + MMP7 + MMP8	28.17	0.69	0.265	4
MMP3 + MMP8 + MMP9	29.35	1.87	0.147	4
MMP3 + MMP7 + MMP8 + MMP9	30.10	2.62	0.101	5

Δ(AIC)—delta Akaike’s information criterion; wt.(AIC)—weight of AIC; k—degree of freedom. MMP 3—metalloproteinase 3; MMP 7—metalloproteinase 7; MMP 8—metalloproteinase 8; MMP 9—metalloproteinase 9.

## Data Availability

Data are contained within article.
